# Editorial: Failures and repairs in human-robot communication

**DOI:** 10.3389/frobt.2025.1583911

**Published:** 2025-03-10

**Authors:** Frank Förster, Patrick Holthaus

**Affiliations:** Robotics Research Group, School of Physics, Engineering and Computer Science, University of Hertfordshire, Hatfield, United Kingdom

**Keywords:** human-robot interaction, conversation analysis (CA), dialogue systems, spoken dialogue systems, speech interfaces, failures, conversational breakdown

## 1 Introduction

This Research Topic arose on the back of the WTF workshop series (Förster et al., 2022; Förster et al., 2023a) that brought together an interdisciplinary group of researchers ranging from roboticists and computational linguists to conversation analysts and cognitive scientists to openly and frankly discuss failures of (robotic) speech interfaces they experienced when deploying these in their studies. Some of the issues discussed in the workshops are elaborated in the contributed articles below, more pointers can be found in the workshop summary article by Förster et al. (2023b).

This Research Topic contributes towards two main objectives: Firstly, we provide a platform for reporting commonly occurring communicative failures in human-robot interaction (HRI). Secondly, this topic aims to highlight the opportunity of potential multi-modal repair mechanisms to render robotic speech interfaces more resilient concerning conversational breakdowns. Hence, we include several articles documenting and analysing such failures to shed light on what is largely an unreported issue experienced by many robotics practitioners. Moreover, this topic also contains articles reporting existing research on conversational repair in HRI and position papers outlining the potential of such mechanisms.

## 2 Contributed articles

Addlesee and Papaioannou point out a number of practical issues linked to spoken dialogue systems (SDS) based on both their own experience as well as existing literature when deploying social robots in real-world settings. They report evidence of people struggling to understand robots due to an insufficient volume of robots’ voices either due to noise in the environment, limited hearing on the part of the human interlocutors or a combination of both. A second, and in some sense symmetrical issue is that robots frequently cannot hear the human interlocutor. This is typically caused by an insufficient number of built-in microphones or a suboptimal placement of these, e.g., microphones being located behind covering materials. Addlesee and Papaioannou further discuss the related problem of ego-noise, that is, noise that is generated by the robot itself, negatively impacting the speech recognition capability of the robot. As the authors emphasize, all of the highlighted issues could be fixed in a relatively straightforward manner if social robots were designed–from the very start–under consideration of their prospective speech capabilities, rather than microphones and speech-related design decisions being integrated and made at a comparatively late design stage.

Galbraith investigate how virtual assistants deal with the interactionally highly relevant and frequent “*huh?*”, an other-initiated, and likely universal repair marker (cf. Dingemanse et al., 2015). They further investigate what repair strategies these assistants utilise when encountering unintelligible speech, and how native speakers judge these different repair strategies. In their study, two different virtual assistants, Google Assistant and Apple’s Siri, are compared across two different languages (English and Spanish). Galbraith finds that neither assistant actively produces “*huh?*” but rather employs more specific repair strategies when confronted with unintelligible speech. The assistants frequently have trouble dealing with a “*huh?*” produced by human users, and some of the repair strategies employed by the two assistants were rated negatively by human judges. While these insights were gained by interacting with virtual assistants, we expect some of these to apply to SDS more generally (cf. Lopez et al., 2022).

Tisserand et al. present a conversation analysis of sequential failures they observed in a large HRI corpus gathered via an in-the-wild study with the Pepper robot that was placed at the entrance of a university library. The failures they found fell frequently into one of four categories: (1) the inability of Pepper’s SDS to distinguish different types of conversational actions involving identical key words, here words associated with greetings; (2) the inability to detect when the human interlocutor takes back the initiative, leading to the robot talking over the human; (3) the failure to detect turn-holding devices; and (4) the SDS’ inability to detect when two conversational actions are produced within the same turn. Tisserand et al. subsequently outline the requirements for future dialogue systems that would need to be fulfilled to avoid these types of failures and review the current state of the technical literature with respect to these requirements. This paper illustrates how conversation analysis can be used to provide concrete guidance for future technical developments.

One work within this topic (Frijns et al.) investigates mistakes in a robot’s knowledge base, in particular those kinds of mistakes that a robot is *not* aware of. The authors present a user study that leverages the human interaction partner to help a robotic system identify and correct its own misconceptions. For that, they initially compare people’s preference for speech or visual communication about the robot’s knowledge base in a sorting scenario finding that a combination thereof is being preferred by participants. Moreover, unplanned mistakes that occurred during this study have been found to not be covered by existing failure taxonomies in the field of human-robot interaction. As a consequence, the authors introduce the concept of a *productive failure* and argue that failures often occur as a result of multiple, intertwined causes. The study further highlighted that mistakes can play an important role for users when familiarising themselves with a robotic system where they frequently test the robot’s limits to better understand its operating principles.

## 3 Conclusion

The articles collated under this Research Topic highlight frequently occurring failures in robotic speech interfaces when these are deployed in-the-wild, many of which may not be observed when these SDS are assessed via benchmark datasets. They provide several concrete recommendations on how to improve both robot and SDS design to reduce the latter’s propensity for failure, and we hope that they will help to guide research efforts to render robotic speech interfaces more resilient when deployed outside of laboratory settings.
